# Editorial: Orchid Genomics and Developmental Biology

**DOI:** 10.3389/fpls.2020.01013

**Published:** 2020-07-10

**Authors:** Jen-Tsung Chen, Katharina Nargar

**Affiliations:** ^1^ Department of Life Sciences, National University of Kaohsiung, Kaohsiung, Taiwan; ^2^ Australian Tropical Herbarium, James Cook University, Cairns, QLD, Australia; ^3^ National Research Collections Australia, Commonwealth Industrial and Scientific Research Organisation (CSIRO), Canberra, ACT, Australia

**Keywords:** orchid, biotechnology, functional genomics, developmental biology, evolution

Orchidaceae is the second largest family of flowering plants with more than 27,000 species inhabiting nearly every habitat worldwide. Orchids exhibit an exceptional morphological and ecological diversity, and are highly valued on the global horticultural market. Orchids possess unique morphological and physiological characteristics, such as highly reduced seeds with an immature embryo, complex flower structures such as the gynandrium and labellum, and have evolved crassulacean acid metabolism and mycoheterotrophy multiple times independently. This range of traits renders orchids prime non-model plants for studying different aspects of evolution through mechanistic studies considering gene function, physiology, and phylogenetic relationships. As summarized below, this Research Topic presents recent advances in orchid biology and consists of 12 publications in the fields of reproductive development, evolution, biotechnology, and photosynthesis.

## Flower Organ Development

The study of transcription factors (TFs), proteins that bind to the regulatory DNA sites of specific genes and are involved in the process of transcription, is an important tool for understanding flower development in orchids. Valoroso et al. studied the expression and interaction of three myeloblastosis (MYB) TFs that are part of a regulatory module, including DIVARICATA (DIV), RADIALIS (RAD), and DIV-and-RAD-Interacting-Factor (DRIF), on flower development of the Italian orchid, *Orchis italica,* native to the Mediterranean region. The results showed higher levels of gene expression in the labellum than in lateral inner tepals of the *O. italica* flower. In addition, yeast two-hybrid analysis revealed that the *Orchis*-MYB TF OitDRIF1 interacts with OitDIV and OitRAD. Hence, the results support the hypothesis that these three MYB TFs have a crucial role in controlling zygomorphy in orchid flowers.

Lai et al. investigated the function of a SHINE-Like TF, namely PeERF1, in several *Phalaenopsis* orchids. The authors conclude that PeERF1 is crucial for the development of labellum epidermis, particularly in the formation of nanoridges.

In model plants, MADS-box TFs have been shown to play crucial roles in diverse developmental processes. Based on a comprehensive review of the literature, Teo et al. summarize reproductive developmental processes in model plants including floral transition and patterning, and discuss unique aspects of orchid floral development such as the modified ABC model and the perianth code.

An overview on orchid flower development is provided by Wang et al. summarizing recent advances in our understanding of floral induction, transition, and flowering in popular orchid genera such as *Dendrobium*, *Oncidium*, and *Phalaenopsis*. The authors propose a hypothetical pathway for flowering regulation in *P. aphrodite* under low ambient temperature, and discuss models of the orchid code and perianth code in *P. equestris* and *O.* “Gower Ramsey”.

The anatomical features of reproductive organs at different developmental stages of the mycoheterotrophic orchid *Pogoniopsis schenckii* was investigated by Alves et al. The authors compared characteristics of the seed coat in *P. schenkii* to photosynthetic species of the family. *P. schenckii* was found to have a seed coat that originates from the nucellar epidermis and not from the outer integument as in the other species.

## Evolution

Family-wide comparative studies on plastid structure and gene loss facilitate important insights into plastid evolution in Orchidaceae. Kim et al. compared structural variation and gene content of 124 complete orchid plastomes and investigated gene loss in orchids within a phylogenomic framework. The authors provide recommendations for additional molecular studies to increase our understanding of plastome evolution, in particular in mycoheterotropic orchids.

The deceptive flowers of Lady slipper orchids (subfamily Cypripedioideae) possess a slipper shaped labellum that traps insects to facilitate pollination. Tsai et al. investigated evolutionary relationships, intrageneric classification, and historical biogeography of the Venus slipper orchids, genus *Paphiopedilum,* based on nuclear (ITS) and three plastid markers (*trnL* intron, *trnL-F*, *atpB-rbcL*) for 78 taxa. The results supported the current intrageneric classification of the genus and provide insights into biogeographic origin and the spatio-temporal evolution of the genus.

Being one of the largest flowering plant families, orchids have evolved a great variety of pollination strategies. Fernández et al. profiled and compared floral transcriptomes of two European orchid genera with different pollination strategies, the sexually deceptive *Ophrys* and *Gymnadenia* with a food-rewarding pollination strategy. Further, phylogenomic analysis was carried out based on transcriptome data to resolve evolutionary relationships within the two genera.

## Biotechnology

In orchids, protocorm-like bodies (PLBs) induced from explants or callus *in vitro* have long been recognized to resemble somatic embryos (SEs) in some species. Based on a molecular study, Chen et al. reported that PLBs do not derive from SEs at least in *P. equestris*. Further, the authors induced SEs by overexpressing *P. aphrodite LEAFY COTYLEDON1* (*PaLEC1*) and suggested that induction of SEs may be a useful tool for clonal propagation of orchids.

Orchids rely on symbiotic relationships with fungi for seed germination and subsequent seedling growth. Sisti et al. characterized fungal communities in roots and fruits of a mycoheterotrophic orchid, *P. schenckii,* and carried out germination trials with non-mycorrhizal endophytic fungi isolated from *P. schenckii* under *in vitro* conditions. The authors observed that germination was stimulated by inoculation of *P. schenckii* seeds with the isolated non-mycorrhizal fungi.

## Photosynthesis

The photosynthetic pathway known as crassulacean acid metabolism (CAM), found in different plant lineages including orchids, is adaptive in arid conditions due to the high water-use efficiency it confers on the plant. Ceusters et al. studied the metabolite and enzyme patterns of starch degradation in the CAM orchid *Phalaenopsis* “Edessa”. Two routes of degradation were assessed: the hydrolytic route (involving cytosolic D-enzyme, plastid D-enzyme, maltase, and β-amylase), and the phosphorolytic route (involving starch phosphorylase) to compare enzyme activities as well as starch degradation rates. Ceusters et al. concluded that the phosphorolytic pathway might be the main route of starch degradation in CAM orchid *Phalaenopsis*.

## Conclusion and Perspectives

This Research Topic on Orchid Genomics and Developmental Biology provides only a snapshot of this exciting and growing field of research. We hope that with the rapid development of molecular tools and approaches, particularly several orchid genomes and hundreds of transcriptomes that are now available together and with high-throughput technologies and integrative multi-omics, emerging biotechnology such as CRISPR gene editing, researchers will be able to further unravel the secrets of orchid biology with emphasis on mechanistic insights into flowering regulation, flower organ development, embryo/protocorm formation and development, phylogenomics, symbiosis, and photosynthetic pathways, in the future.

We greatly appreciate the invaluable contribution of all authors and reviewers as well as the efforts of Chief Editor Neelima Roy Sinha of Frontiers in Plant Science: Plant Development and EvoDevo.

## Author Contributions

J-TC and KN drafted the manuscript. All authors contributed to the article and approved the submitted version.

## Conflict of Interest

The authors declare that the research was conducted in the absence of any commercial or financial relationships that could be construed as a potential conflict of interest.

